# Isolation and characterization of equine native MSC populations

**DOI:** 10.1186/s13287-017-0525-2

**Published:** 2017-04-18

**Authors:** Cristina L. Esteves, Tara A. Sheldrake, Simone P. Mesquita, Juan J. Pesántez, Timothy Menghini, Lucy Dawson, Bruno Péault, F. Xavier Donadeu

**Affiliations:** 1 The Roslin Institute, University of Edinburgh, Edinburgh, UK; 2 Centre for Regenerative Medicine, University of Edinburgh, Edinburgh, UK; 3 Orthopaedic Hospital Research Centre, University of California, Los Angeles, CA USA; 4 The Roslin Institute, University of Edinburgh, Easter Bush, Midlothian, EH25 9RG UK

**Keywords:** Pericyte, Equine, Horse, Adventitial cells, CD146, CD34, Adipose tissue

## Abstract

**Background:**

In contrast to humans in which mesenchymal stem/stromal cell (MSC) therapies are still largely in the clinical trial phase, MSCs have been used therapeutically in horses for over 15 years, thus constituting a valuable preclinical model for humans. In human tissues, MSCs have been shown to originate from perivascular cells, namely pericytes and adventitial cells, which are identified by the presence of the cell surface markers CD146 and CD34, respectively. In contrast, the origin of MSCs in equine tissues has not been established, preventing the isolation and culture of defined cell populations in that species. Moreover, a comparison between perivascular CD146^+^ and CD34^+^ cell populations has not been performed in any species.

**Methods:**

Immunohistochemistry was used to identify adventitial cells (CD34^+^) and pericytes (CD146^+^) and to determine their localization in relation to MSCs in equine tissues. Isolation of CD34^+^ (CD34^+^/CD146^–^/CD144^–^/CD45^–^) and CD146^+^ (CD146^+^/CD34^–^/CD144^–^/CD45^–^) cell fractions from equine adipose tissue was achieved by fluorescence-activated cell sorting. The isolated cell fractions were cultured and analyzed for the expression of MSC markers, using qPCR and flow cytometry, and for the ability to undergo trilineage differentiation. Angiogenic properties were analyzed in vivo using a chorioallantoic membrane (CAM) assay.

**Results:**

Both CD34^+^ and CD146^+^ cells displayed typical MSC features, namely growth in uncoated tissue culture dishes, clonal growth when seeded at low density, expression of typical MSC markers, and multipotency shown by the capacity for trilineage differentiation. Of note, CD146^+^ cells were distinctly angiogenic compared with CD34^+^ and non-sorted cells (conventional MSCs), demonstrated by the induction of blood vessels in a CAM assay, expression of elevated levels of VEGFA and ANGPT1, and association with vascular networks in cocultures with endothelial cells, indicating that CD146^+^ cells maintain a pericyte phenotype in culture.

**Conclusion:**

This study reports for the first time the successful isolation and culture of CD146^+^ and CD34^+^ cell populations from equine tissues. Characterization of these cells evidenced their distinct properties and MSC-like phenotype, and identified CD146^+^ cells as distinctly angiogenic, which may provide a novel source for enhanced regenerative therapies.

## Background

Other than humans, horses are the species in which interest in the use of mesenchymal stem/stromal cells (MSCs) for regenerative medicine has been highest [1–8]. Similar to humans, adipose tissue and bone marrow have been the MSC sources of choice in the horse, due to their relative ease of access and the number of putative stem cells they contain [2, 9–13]. Indeed, MSCs have been safely applied clinically to horses for well over a decade, now being an integral component of equine health care worldwide which has an estimated economic impact in the United States and the United Kingdom of $102 billion and $10 billion per annum, respectively. Importantly, given the similarities in the types of natural injuries suffered by both humans and horses, the US Food and Drug Administration [14] has approved the use of horses as a preclinical model for musculoskeletal conditions.

In regenerative medicine, cell preparations are applied either as crude extracts or following expansion in culture to attain the desired amount of clinical cells. However, these preparations are heterogeneous in nature, containing small and variable amounts of true stem cells in addition to other, nonprecursor cell types which upon transplantation into patients may reduce therapeutic efficacy. There is therefore a real need to identify specific subsets of cells that have intrinsic regenerative capacity, and to develop strategies to robustly isolate, expand in culture, and characterize these cells. However, there is a fundamental lack of understanding of the precise identity and defining features of MSCs in equine tissues, which has thwarted efforts to selectively and efficiently identify, harvest, and expand these cell subpopulations in culture.

Studies in humans have identified two distinct perivascular cell subpopulations, pericytes in capillaries and microvessels [15, 16] and adventitial cells in arteries and veins [17], as in vivo counterparts of MSCs [18, 19]. Pericytes and adventitial cells were isolated based on the presence of the cell surface markers CD146 and CD34, respectively, and were shown to maintain a typical MSC phenotype in culture. Although tissue regeneration potential has been demonstrated for each individual cell subpopulation [20–22], the properties of CD146 and CD34 cells have not been compared directly.

Using fluorescence-activated cell sorting (FACS), we report the isolation, culture, and characterization of equine CD34^+^/CD146^–^/CD144^–^/CD45^–^ (adventitial cells) and CD146^+^/CD34^–^/CD144^–^/CD45^–^ (pericytes) fractions—for simplicity, referred to hereafter as CD34^+^ and CD146^+^, respectively. Both cell types displayed an MSC phenotype, although comparison with non-sorted cells (conventional cultured MSCs) revealed differences in expression of cell surface markers and angiogenic genes. Remarkably, CD146^+^ cells were distinctly able to promote angiogenesis in vivo, highlighting their potential for tissue regeneration.

## Methods

### Samples

Tissue samples were obtained from a total of 15 adult horses immediately post mortem at the School of Veterinary Studies of the University of Edinburgh. All animal procedures were carried out according to the UK Home Office Animals (Scientific Procedures) Act 1986 with approval by the Ethical Review Committee, University of Edinburgh, 60/4207. Adipose tissue was kept for up to 24 h at 4 °C [23] before extraction of the stromal vascular fraction (SVF).

### Immunohistochemistry

Frozen samples, processed immediately after tissue harvesting, were cut in a Leica CM1900 cryostat. For staining, tissues were fixed in ice-cold acetone:methanol (50:50) and antibodies were prepared in antibody diluent (003118; Invitrogen-Thermo Fisher Scientific, Paisley, UK). After staining, slides were mounted in Fluoroshield with 4′,6-diamidino-2-phenylindole (DAPI;F6057; Sigma, St Louis, MO, USA). Primary antibodies used were CD146-FITC (MCA2141F; AbD Serotec-BioRad, Kidlington, UK), CD144 (AHP628Z; AbD Serotec-BioRad), NG2 (MAB2585; R&D Systems, Minneapolis, MN, USA), αSMA (ab5694; AbCam, Cambridge, UK), CD29 (303015; BioLegend, San Diego, CA, USA), CD44 (AbD Serotec-BioRad), and CD34 (21270341S; ImmunoTools, Friesoythe, Germany). Isotype controls were IgG1-FITC (MCA928F; AbD Serotec-BioRad), IgG1 (MAB002; R&D Systems), IgG1κ (400101; BioLegend), and IgG1 (21335011; ImmunoTools), all raised in mouse; and rabbit IgG (PRABP01; AbD Serotec-BioRad). Secondary antibodies were conjugated to AF488 (A11029), AF568 (A110037), and AF568 (A10042), all from Invitrogen-Thermo Fisher Scientific. Micrographs were produced using a Zeiss LSM710 confocal or Leica DMLB fluorescent microscope.

### Cell extraction and culture

Extraction of the SVF from adipose tissue involved mincing the tissue with scissors and digestion for 45 min with collagenase II (1 mg/ml; Gibco-Thermo Fisher Scientific)/BSA (3.5%) at 37 °C under agitation (100 rpm). Collagenase activity was stopped by addition of DMEM with 20% FBS (Thermo Fisher Scientific) and the lipid layer was removed after separation by gravity. Samples were filtered and spun down to obtain the SVF, which was further washed in PBS/FBS (5%) and treated with red cell lysis buffer (Sigma-Aldrich). Cells were then stained with appropriate antibodies for flow analysis or sorting, or seeded for expansion at 5000 cells/cm^2^ in EGM-2 (Lonza, Slough, UK) which was replaced by DMEM/FBS (20%) at the first cell passage. The doubling time of non-sorted, CD34^+^, or CD146^+^ cultured cells was calculated using the formula:

$$ \mathrm{D}\mathrm{T}= T \ln 2/ \ln \left({X}_{\mathrm{e}}/{X}_{\mathrm{b}}\right), $$

where *T* is the incubation time in days, *X*_b_ is the cell number at the beginning of the incubation time, and X_e_ is the cell number at the end of the incubation time. To determine colony-forming unit fibroblasts (CFU-F), cells were seeded at a density of 1, 5, and 25 cells/cm^2^ and allowed to grow for 12 days. Colonies were visualized by staining with crystal violet (0.5%) and counted. To obtain endothelial cells, blood vessel sections were washed with PBS and then filled with collagenase II solution (1 mg/ml) before both ends of the vessel were ligated. Following 45-min incubation at 37 °C, endothelial cells were recovered and were cultured in EGM-2 endothelial cell medium.

### Flow cytometry and FACS

In order to isolate cell subpopulations, SVFs were stained for 1 h on ice using primary antibodies against CD144 (AHP628Z; AbD Serotec-BioRad), CD45-FITC (MCA832F; BioRad), CD146-AF647 (MCA2141A647; AbD Serotec-BioRad), and CD34-PE (4H11[APG]; ImmunoTools) followed by incubation for 30 min on ice with AF405-conjugated secondary antibody (ab175654; AbCam). Isotype controls used (raised in mouse) were IgG1-FITC (MCA928F; AbD serotec-BioRad), IgG1-AF647 (MCA928A647, AbD serotec-BioRad), and IgG1-PE (21275514; ImmunoTools). Fluorescence minus one (FMO) for CD146 was obtained with a mix of antibodies against CD144, CD45, and CD34, and the mix for CD34 FMO included CD144, CD45, and CD146. DAPI was added to the samples 5 min before flow analysis and was used to determine cell viability. Cells were sorted on a BD FACSAria Fusion (BD Biosciences, San Jose, CA, USA).

In order to analyze the immunophenotype of cultured cells, non-sorted, CD34^+^, and CD146^+^ cells were harvested using accutase (SLBN3817V; Sigma). The staining protocol was similar to that already described for FACS and included primary antibodies to CD29 (303001; BioLegend), CD44 (MCA1082GA; AbD serotec-BioRad), CD90 (554895; BD Biosciences), and CD105 (MCA1557T; AbD serotec-BioRad), as well as the isotype controls mouse IgG1κ (400101; BioLegend) and mouse IgG1 (MAB002: R&D Systems). Secondary antibody was AF488-conjugated (A11029; Invitrogen-Thermo Fisher Scientific), and Sytox blue (S34857; Thermo Fisher Scientific) was used to determine cell viability. Samples were run on a BD LSR Fortessa (BD Biosciences) and data were analyzed using BD FACSDiva software version 8.0.1 or FlowJo (LLC, Ashland, OR, USA).

### RNA extraction and gene expression analyses

Cultured cells or freshly collected SVFs were harvested in Trizol (15596-026; Invitrogen-Thermo Fisher Scientific) and the concentration of total RNA was measured using Nanodrop. RNA was reverse transcribed with SuperScript III (18080-044; Invitrogen-Thermo Fisher Scientific) and qPCR was performed using the SensiFAST SYBR Lo-ROX kit (BIO-94020; Bioline, London, UK) in a Stratagene machine. Primers were either designed by us for CD144 (5′-TCTGCAGGACATCAATGACAAC-3′ and 5′-CTTCAG GCACGGCAAATACG-3′) and 18S (5′-GCTGGCACCAGACTTG-3′ and 5′-GGGGAATCAGGGTTCG-3′) or were based on previously reported sequences [24]. Data were analyzed with MxPro software and normalized to 18S.

### Cell differentiation

Non-sorted, CD34^+^, and CD146^+^ cultured cells were differentiated into adipocytes, chondrocytes, and osteocytes. Briefly, for adipocyte differentiation, cells were grown until 70% confluency [25, 26] and induced in the presence of dexamethasone (1 μm, D4902; Sigma Aldrich), 3-isobutyl-1-methylxanthine (0.5 mM, 15879; Sigma Aldrich), insulin (10 μg/ml, 19278; Sigma Aldrich), indomethacin (100 μM, I8280; Sigma Aldrich), rabbit serum (7%), and fetal bovine serum (3%). Cells were differentiated for 5 days with medium being changed twice. Chondrogenesis and osteogenesis were performed using StemPro differentiation kits following the manufacturer’s instructions for 18 days (A10071-01 and A10072-01, respectively; Life technologies-Thermo Fisher Scientific). Adipocytes were stained with Oil red O (O0625; Sigma-Aldrich) as described before [27]. In brief, cells were first fixed in paraformaldehyde (PFA; 4%) for 3 min, rinsed with 60% isopropanol, and stained with Oil red O (0.1%) for 10 min and then rinsed with isopropanol again and washed with water. Chondrocyte pellets were embedded in Histogel (HG-4000; Invitrogen-Thermo Fisher Scientific) and paraffined, and then cut at 8 μm thickness. Slides were dewaxed and rehydrated. Alcian blue solution (1%) was added to the slide and incubated overnight. After washing, Neutral red (1%) solution was added and absolute ethanol and xylene were used to dehydrate the slides that were mounted with pertex. For osteocyte staining, cells were washed with PBS, fixed with PFA (4%) for 15 min, and stored in PBS at 4 °C. After washing the cells with water, Alizarin Red S (A5533; Sigma-Aldrich) was added. The plate was incubated at room temperature in the dark for 10 min and washed with water. Alkaline phosphatase activity was determined in differentiated cells using a commercial kit (86R-1KT; Sigma-Aldrich) according to the manufacturer’s specifications. Briefly, cells were washed twice with PBS, then fixed with PFA-citrate acetate buffer for 30 sec and washed again with PBS, to which the alkaline–dye mixture was added for the reaction to proceed with wells protected from light, and then washed with water. Negative controls were produced with nondifferentiated cells in culture and micrographs were taken on an Axiovert 25 inverted microscope (Zeiss, Oberkochen, Germany).

### Angiogenesis assays

The chorioallantoic membrane (CAM) assay was used to assess in vivo angiogenic potential of non-sorted cells and sorted CD34^+^ and CD146^+^ cells. Fertilized eggs (NovoGen, Le Foeil, France) were cleaned with ethanol (70%) and incubated at 37 °C in 60% humidity. Three days later, 2 ml of albumen was aspirated from the egg with a syringe and a window was created which was covered again and returned to incubation. On day 8 of incubation, polypropylene rings were placed on the CAM and cells (75,000 cells) or PBS were placed in the center of the ring, as described previously [28, 29]. The eggs were incubated for a further 2 days, when images from each ring were taken using the Zeiss AXIO ZoomV16 microscope at 40×. Binary images were generated by Image J and angiogenesis was quantified using AngioSys2.0 software (Cellworks, Buckingham, UK).

For in vitro angiogenesis experiments, CD146^+^ and endothelial cells were stained with PKH26 (20 μM) and PKH67 (20 μM) (both from Sigma-Aldrich), producing red and green fluorescence signals, respectively. Once labeled, cells were resuspended in EGM-2 medium and seeded on ibiTreat μ-Slides (IB-81506; Thistle Scientific) coated with matrigel. Pictures were taken using a Zeiss Live Cell Observer/Deconvolution system.

### Statistical analysis

Results are shown as mean ± standard error of the mean, and were analyzed by one-way or two-way ANOVA followed by comparison among means with Tukey test using GraphPad Prim 6.0 software (GraphPad Software, La Jolla, CA, USA). Significance was set at *p* < 0.05.

## Results

### CD146 and CD34 mark perivascular cells and colocalize with MSC markers in equine tissues

**Fig. 1:**
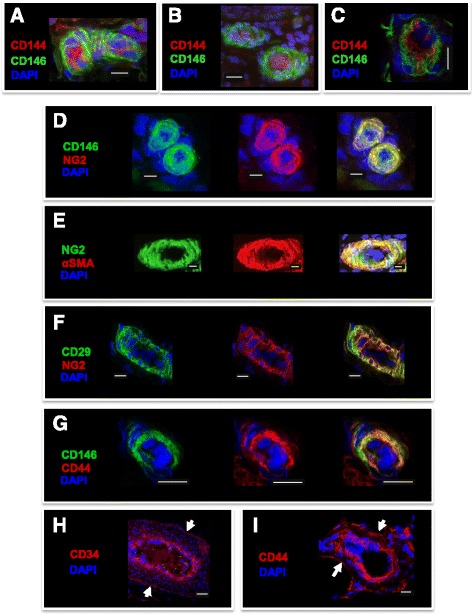
Immunohistochemistry of pericyte, adventitial cell, and MSC markers in equine tissues. **a**–**c** Pericytes stained for CD146 surrounding CD144^+^ endothelial cells in adipose tissue (**a**), testis (**b**), and skeletal muscle (**c**). **d**, **e** Dual staining (*right panel*) with the pericyte markers NG2 and CD146 (**d**) or NG2 and αSMA (**e**) in adipose tissue and testis, respectively. **f**, **g** Colocalization (*right panel*) of the MSC and pericyte markers CD29 and NG2 (**f**) and CD44 and CD146 (**g**) in adipose tissue, respectively. **d**–**g** Individual staining is shown (*left* and *middle panels*). **h**, **i** Immunodetection of the adventitial cell marker CD34 (**h**) and the MSC marker CD44 (**i**) in the outer layer of blood vessels (*arrows*) in adipose tissue. 4′,6-Diamidino-2-phenylindole (*DAPI*) was used to stain cell nuclei. *White scale bars* = 10 μm

We began by testing the cross-reactivity of cell surface marker antibodies in equine tissues. In human tissues, CD146 (MCAM, Muc18) has been commonly used to identify pericytes surrounding small blood vessels [16, 30]. Similarly, in equine tissues including adipose, testis, and skeletal muscle, CD146 had an abluminal location to the endothelial marker CD144 (VE-cadherin); importantly, CD144 was not present in CD146^+^ cells, indicating that these corresponded to pericytes (Fig. 1 a–c). Other antigens known to be expressed by pericytes, namely NG2 (CSPG4), αSMA (ACTA1), and CD146, colocalized in the tissues analyzed (Fig. 1d, e). Moreover, the MSC markers CD29 and CD44 were also detected perivascularly, costaining with NG2 and CD146, respectively (Fig. 1f, g). Adventitial cells were identified by CD34^+^ staining in the outer layer of larger blood vessels, which followed a pattern similar to that of the MSC marker CD44 (Fig. 1h, i). Overall, these results showed that pericytes and adventitial cells colocalize perivascularly with MSC markers in horse tissues, consistent with the notion in the human that perivascular cells give rise to MSCs in culture [15, 17].

### Isolation of CD146^+^ and CD34^+^ cells from equine adipose tissue

**Fig. 2:**
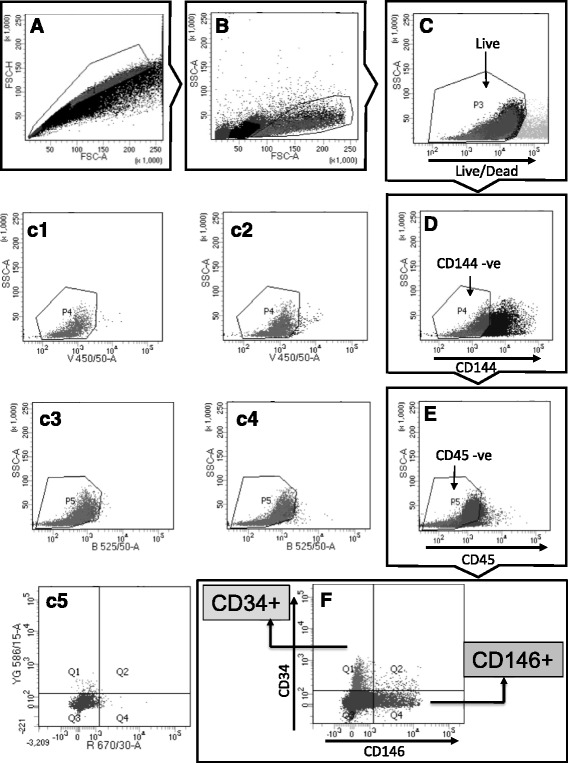
Isolation of CD34^+^ and CD146^+^ cells from adipose SVF. **a**, **b** Events displayed as FSC-A vs FSC-H to select singlets (**a**) were further gated to exclude cell fragments or noncellular material (**b**). **c**–**e** Sequence of plots showing selection of DAPI-negative, CD144(AF405)-negative, and CD45(FITC)-negative cells to obtain live, endothelial-negative, and hematopoietic-negative fractions, respectively. (*c1–c4*) Dot-plots for CD144 (*c1*, *c2*) and CD45 (*c3*, *c4*) controls showing unstained cells (*c1*, *c3*) and secondary antibody conjugated to AF405 (*c2*) or isotype conjugated to FITC (*c4*). **f** Double-plot displaying CD34^+^ (Q1) and CD146^+^ (Q4) cell subpopulations and respective isotype controls (*c5*) conjugated to PE and AF647, respectively. Filters used: 450/50 for AF405, 525/50 for FITC, 586/15 for PE, and 670/30 for AF647. *FSC-A* forward scatter area, *FSC-H* forward scatter height, *SSC-A* side scatter area

There are no reports on the isolation of perivascular cells in the horse. Extraction of the stromal vascular fraction (SVF) from adipose tissue by standard collagenase digestion resulted in an adipocyte-free preparation (around 300,000 cells/g tissue) containing different cell types including many erythrocytes. Thus, an essential step in the preparation of samples for FACS was incubation with red blood cell lysis buffer, because high numbers of erythrocytes could interfere with antibody staining, flow analysis, and cell sorting. Selection of an antibody panel for FACS was based on IHC validation (Fig. 1) as well as optimization by flow cytometry using equine adipose, testes, and peripheral blood mononuclear cell samples (data not shown). FACS of CD34^+^ and CD146^+^ subpopulations from adipose SVF extracts sequentially involved: selection of single events (single cells) plotted as forward scatter area vs forward scatter height (FSC-A vs FSC-H; Fig. 2A), visualized in the dot-plot of FSC-A vs side scatter area (SSC-A; Fig. 2B); selection of DAPI-negative (live) cells (Fig. 2C); sorting out of CD144^+^ endothelial cells (Fig. 2D); gating of CD45-FITC-negative cells (Fig. 2E); and selection of cells positive for CD34-PE and CD146-AF647 (Fig. 2F) to separately obtain CD45^–^/CD144^–^/CD146^–^/CD34^+^ (CD34^+^ cells; 0.6 ± 0.4% of total) and CD45^–^/CD144^–^/CD34^–^/CD146^+^ cells (CD146^+^ cells; 1.9 ± 0.7% of total). Antibody isotypes or AF405-linked secondary antibodies were used as controls (Fig. 2c2, c4, c5). CD34^+^ and CD146^+^ cells were then cultured and characterized.

### CD146^+^ and CD34^+^ cells show similar growth in culture

**Fig. 3:**
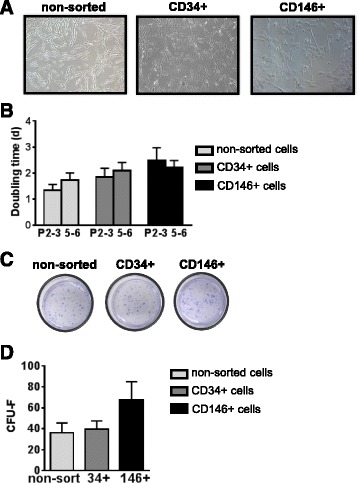
Growth of sorted cells in culture. **a** Micrographs showing similar morphology for non-sorted, CD34^+^, and CD146^+^ cells grown in DMEM supplemented with 20% FBS and passaged at a density of 5000 cells/cm^2^. **b** Doubling times (days) for non-sorted, CD34^+^, and CD146^+^ cells at passages 2–3 (*P2-3*) and 5–6 (*P5-6*). *n* ≥ 5; mean ± SEM. **c**, **d** Colonies obtained from cells grown at low density (5 cells/cm^2^) were stained with crystal violet (**c**) and CFU-F were counted (**d**). *n* ≥ 3; mean ± SEM. *CFU-F* colony-forming unit fibroblasts

Non-sorted cells (conventional MSCs) and sorted CD34^+^ and CD146^+^ cells showed similar morphology in culture (Fig. 3a). Cells were passaged and seeded at a density of 5000 cells/cm^2^, producing similar doubling times between passages 2 and 6 (Fig. 3b). CFU-F (Fig. 3c) were quantified by seeding cells at low densities (1, 5, and 25 cells/cm^2^) and staining colonies with crystal violet 12 days later. In our hands, a seeding density of 5 cells/cm^2^ was optimal and was used for CFU-F quantification, with no significant differences being found between the three cell types (Fig. 3d).

### Expression of cell surface markers by CD146^+^ and CD34^+^ cells in culture

**Fig. 4:**
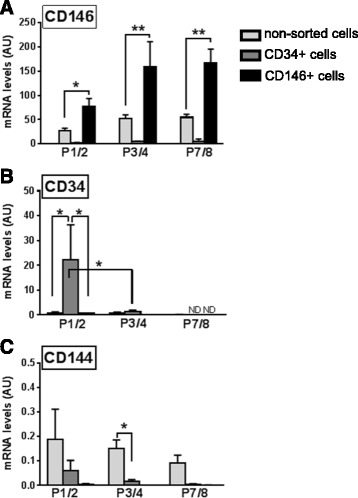
Gene expression analysis of non-sorted, CD34^+^, and CD146^+^ cultured cells. mRNA levels were quantified by qPCR using primers for **a** CD146 (pericyte marker), **b** CD34 (adventitial cell), and **c** CD144 (endothelial cells) in non-sorted (*light gray*), CD34^+^ (*dark gray*) and CD146^+^ (*black*) cells at passage 1 or 2 (*P1/2*), 3 or 4 (*P3/4*), and 7 or 8 (*P7/8*). *n* = 3–6; mean ± SEM. *p < 0.05, **p < 0.001 between means. *AU* arbitrary units, *ND* not detected

Based on qPCR analyses, CD146^+^ cells expressed the highest levels of CD146 throughout culture, around 3-fold higher than non-sorted cells (*p* < 0.05; Fig. 4a), showing that the isolation process was effective. CD146 mRNA was detected in some CD34^+^ cell samples but at markedly reduced levels compared with CD146^+^ cells (27-fold lower in average), which may correspond to a transitional minority subpopulation between pericyte and adventitial cells as described in humans [30]. Expression of CD34 was variable and substantial only in CD34^+^ cells at passages 1/2 and then decreased (Fig. 4b; *p* < 0.05), as is known to occur in cells in culture [31, 32]. CD34 was absent in the CD146^+^ cells, except for very low levels in one of the samples. CD144 was detected in all non-sorted samples (Fig. 4c) at lower values than in the initial SVF crude extract (data not shown). CD144 was expressed at much lower levels (around 13-fold) in CD34^+^ cells and was occasionally detected, at negligible levels, in CD146^+^ cells. CD45 was not detected except in one of the non-sorted preparations at passages 1/2 (data not shown), an observation that we have occasionally made in other non-sorted samples and which has also been reported by others [33].

### CD146^+^ and CD34^+^ cells express MSC markers

**Fig. 5:**
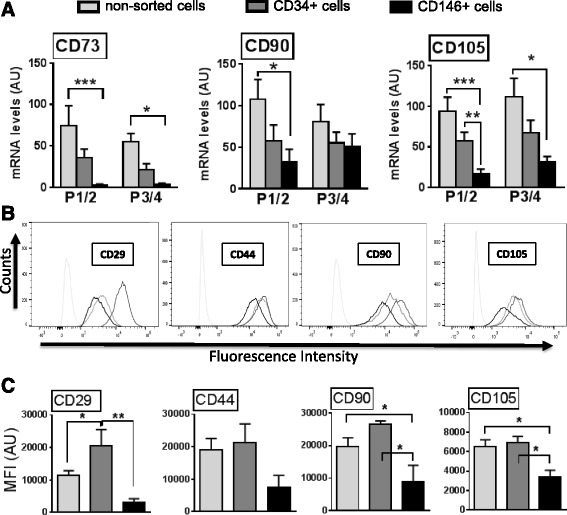
Isolated CD146^+^ and CD34^+^ cells express MSC markers. qPCR measurements (**a**) of CD73 (*left*), CD90 (*middle*), and CD105 (*right*) of non-sorted (*light gray*), CD34^+^ (*dark gray*), and CD146^+^ (*black*) cells analyzed at passage 1 or 2 (*P1/2*) and 3 or 4 (*P3/4*). Flow cytometry histograms (**b**) displaying fluorescence intensity (AF488 conjugated to the secondary antibody) vs event counts (corresponding to number of cells) and showing displacement of non-sorted (*light gray*), CD34^+^ (*dark gray*), and CD146^+^ (*black*) cultured cells stained with antibodies CD29, CD44, CD90, and CD105 in comparison with the isotype controls (*very light gray*, on *left*). Data were obtained from P3 to P5. **c** Quantification of the flow signals depicted in bar graphs as mean fluorescence intensity (*MFI*) of non-sorted (*light gray*), CD34^+^ (*dark gray*), and CD146^+^ (*black*). *n* ≥ 3; mean ± SEM. *p < 0.05, **p < 0.001, ***p < 0.0001 between means. *AU* arbitrary units

qPCR analysis demonstrated that MSC markers were present in both CD34^+^ and CD146^+^ cells in culture (Fig. 5). Indeed, CD73, CD90, and CD105 mRNA was detected in both cell types as well as in non-sorted cells up to at least passage 4 (Fig. 5a). Expression of these genes was lower in CD146^+^ cells than in non-sorted cells at all passages (*p* < 0.05), except for CD90 at passages 3/4. Moreover, apart from higher (*p* < 0.05) expression of CD105 in CD34^+^ cells than CD146^+^ cells at passages 1/2, there were no differences in expression of the three MSC markers between CD34^+^ and the other two cell types.

In agreement with the qPCR results, all MSC markers tested by flow cytometry (CD29, CD44, CD90, and CD105) were present in CD34^+^ and CD146^+^ cells (Fig. 5b). Importantly, for all markers the antibody-positive cells produced distinct peaks from the isotype controls, indicating that the great majority of the cells in the samples expressed the respective maker. However, differences were observed in mean fluorescence intensity (MFI), suggesting differences in antigen level between cell types (Fig. 5c). In general, MFI values for MSC markers were lower in CD146^+^ cells than in non-sorted and CD34^+^ cells, although these differences were not significant in the case of CD44.

### CD146^+^ and CD34^+^ cells are multipotent

**Fig. 6:**
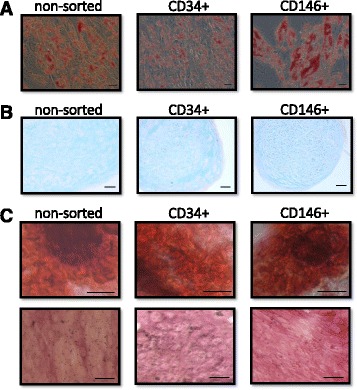
Isolated CD146^+^ and CD34^+^ cells are multipotent. Micrographs showing differentiation of non-sorted (*left*), CD34^+^ (*middle*), and CD146^+^ cells (*right*) into adipocytes (**a**), chondrocytes (**b**), and osteocytes (**c**). **a** Adipocytes were stained with Oil red O, showing the lipid content in the cells (*red*). **b** Histological sections (8 μm) of differentiated chondrocyte pellets were stained with Alcian *blue* to detect glycosaminoglycans and counterstained with Neutral *red*. **c** Alizarin Red S and the alkaline phosphatase activity assay were used to stain osteocytes differentiated for 19 days, resulting in staining of calcium deposits (*red*, *upper row* and *purple*, *lower row*, respectively). *Black scale bars* = 50 μm

Similar to non-sorted cells, both CD34^+^ and CD146^+^ populations were capable of trilineage differentiation (Fig. 6). Upon culture in adipogenic media, all three cell types became round in morphology and accumulated lipid vesicles, as shown by Oil red O staining in the cytoplasm (Fig. 6a). Chondrogenesis and osteogenesis were also observed in the three cell types, with Alcian blue coloring glycosaminoglycanss in chondrocyte pellets counterstained with Neutral red (Fig. 6b). Osteocytes displayed staining of calcium deposits by Alizarin Red and were also alkaline phosphatase-positive (Fig. 6c). These results indeed show that multipotency is maintained by CD146^+^ and CD34^+^ cells in culture.

### Equine CD146^+^ cells promote angiogenesis

**Fig. 7:**
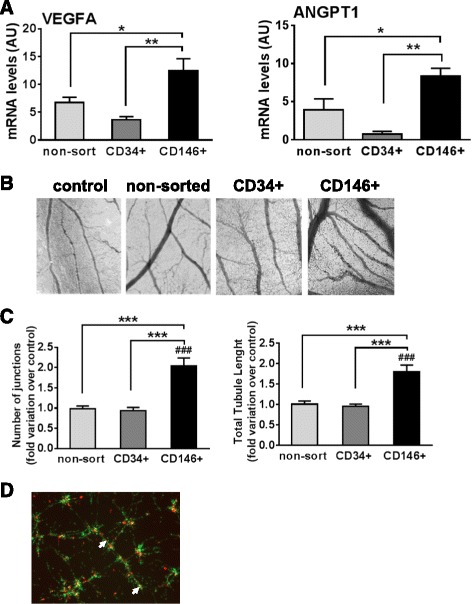
CD146^+^ cells are angiogenic. **a** Transcript levels of angiogenic factors VEGFA and ANGPT1 in non-sorted (*light gray*), CD34^+^ (*dark gray*), and CD146^+^ (*black*) cells, quantified by qPCR. **b** CAM micrographs obtained at 40× magnification showing formation of blood vessel plexus at 2 days following incubation with PBS control (*left*), non-sorted (*middle left*), CD34^+^ (*middle right*), and CD146^+^ (*right*) cells. **c** Quantification of the CAM blood vessel plexus using AngioSys2.1 software as fold variation in blood vessel branching (*left*) and blood vessel total length (*right*) over PBS-treated controls. Non-sorted (*light gray*), CD34^+^ (*dark gray*), and CD146^+^ (*black*) cells. **d** Micrographs of in vitro cocultures of equine endothelial (*green*, labeled with PKH26; 20 μM) and CD146^+^ (*red*, labeled with PKH67; 20 μM) cells seeded on matrigel, showing close apposition of CD146^+^ cells to the endothelial network (*white arrows*). *n* ≥ 3 for qPCR, *n* ≥ 12 for CAM experiments; mean ± SEM. *p < 0.05, **p < 0.001, ***p < 0.0001, differences between different cell types; ###*p* < 0.0001, difference between CAM incubated with CD146^+^ cells and with PBS (control). *AU* arbitrary units

Angiogenesis is crucial in tissue regeneration and can be reportedly stimulated by MSCs [34]. For this reason, we determined the relative expression of the angiogenic factors VEGFA and ANGPT1 by CD34^+^, CD146^+^, and non-sorted cells, as well as the capacity of each cell type to promote angiogenesis in vivo (Fig. 7). The three cell types expressed VEGFA and ANGPT1, but levels of the two genes were significantly higher in CD146^+^ cells than in non-sorted or CD34^+^ cells (Fig. 7a). We then tested angiogenic activity using the chick embryo chorioallantoic membrane (CAM) assay, a method that has been extensively used for angiogenic assays in tumor [28] and stem cell [34–36] studies. The CAM is easily accessible and allows quantification of angiogenesis in a large number of samples simultaneously. In agreement with qPCR data, we found that CD146^+^ cells distinctly promoted angiogenesis in vivo, as shown by significant increases (relative to PBS control) in blood vessel branching and total vessel length in membranes exposed to CD146^+^ cells compared with non-sorted and CD34^+^ cells (Fig. 7b, c). In addition, coculture experiments (Fig. 7d) demonstrated the association between CD146^+^ cells and endothelial cells on matrigel, suggestive of network stabilization by CD146^+^ as occurs naturally in tissues, and indicating that these cells maintained their native properties in culture.

## Discussion

Despite the enormous interest in the use of MSC therapies in both humans and horses, and the impressive amount of literature devoted to this topic over the last decade, cell heterogeneity in clinical MSC preparations has significantly hindered the full exploitation of these therapies. The identification of two distinct perivascular cell subpopulations, based on the expression of CD146 and CD34, as precursors of MSCs in human tissues [15, 17] provided a significant step forward toward new therapies with increased clinical efficacy. However, studies using defined cell subpopulations (as opposed to traditional heterogeneous MSC preparations) in the horse are lacking, largely a consequence of the difficulty in translating cell isolation procedures developed for human cells to other species and the limited availability of species-specific antibodies. Here, we have successfully established for the first time a methodology to isolate perivascular CD34^+^/CD146^–^/CD45^–^/CD144^–^ and CD146^+^/CD34^–^/CD45^–^/CD144^–^ populations in the horse. We report the successful expansion of these cells in culture as well as the characterization of their properties relative to non-sorted MSC populations.

The observation that CD146 and CD34 have a perivascular location in different equine tissues which coincides with that of established MSC markers is consistent with the notion that pericytes and adventitial cells are native MSC precursors [15, 17, 37], thus extending previous observations in humans to the horse. Our finding that CD146 is robustly maintained by sorted CD146^+^ cells in culture is in agreement with two previous PCR [38] and flow cytometry [39] studies reporting the presence of CD146 in equine MSCs. In contrast, expression of CD34 in CD34^+^ cells was variable and quickly decreased upon culture, as reported for other cells [31, 32]. This may account in part for the fact that CD34 has only occasionally been detected by PCR in equine MSCs [38, 40], and for the difficulty so far in identifying CD34 antibodies cross-reacting with equine cells. Indeed, in the present study only one of the five CD34 antibodies tested could be used successfully with equine samples.

CD34^+^ and CD146^+^ populations displayed typical MSC properties, namely adherence to uncoated tissue-culture dishes, inclusion of CFU-F, and trilineage differentiation, in addition to expressing classical MSC markers both in tissues and during culture. Importantly, flow cytometry analyses indicated that these markers were present in almost all cells within the two sorted subpopulations, although corresponding MFI and mRNA levels were in general lower in CD146^+^ cells than in the other cell types analyzed. This could be attributed to loss of cell surface marker expression during the time in culture. Another possibility is that cell contaminants such as hematopoietic and/or endothelial cells, which can also express MSC markers [41–45], contributed to the relatively higher expression of these markers, particularly in non-sorted cell cultures. Consistent with this assumption, the single non-sorted cell sample with detectable CD45 mRNA also displayed the highest values for CD90 and CD105 and, in addition, the endothelial marker CD144 was also expressed by non-sorted cells in culture. Regardless of the reasons for these differences, the results indicate that CD146^+^ cells are distinct from non-sorted and CD34^+^ cells in equine adipose tissue.

Angiogenesis has a critical contribution to tissue regeneration by allowing supply of oxygen, nutrients, and immune cells and clearing of metabolic products [46, 47]. Because of their anatomical location, pericytes and adventitial cells are uniquely positioned to regulate angiogenesis both by direct contact with endothelial cells and by paracrine production of angiogenic factors [23, 48, 49]. Using the CAM model, we showed that, under our experimental conditions, CD146^+^ cells, but not non-sorted or CD34^+^ cells, were able to promote angiogenesis in vivo, presumably due in part to their higher expression of the angiogenic factors VEGFA and ANGPT1. In the horse, two publications [50, 51] reported on the angiogenic activity of MSCs, specifically on the stimulation of vascular network formation by peripheral blood-derived MSCs in culture [50] and the proangiogenic effects of adipose MSC-derived membrane vesicles in rat aortic rings and scratch assays [51]. However, in those reports the effects of MSCs in vivo were not determined and, in addition, the specific subpopulations involved in the responses observed in vitro were not identified. Limited studies in humans have gone further to determine the angiogenic effects of isolated CD146^+^ or CD34^+^ populations in vivo using animal models. In particular, human CD146^+^ cells were shown to promote angiogenesis after transplantation into SCID/mdx mouse muscle [52] and in SCID mouse ischemic myocardium [22]. Moreover, improvement of angiogenesis in ischemia was reported in response to CD34^+^/CD31^–^ cells obtained from human saphenous vena; however, other cell types in these preparations could have contributed to the effects observed [53]. Importantly, the effects of CD146^+^ and CD34^+^ on angiogenesis had so far not been directly compared in any species. Our results using the equine model show CD146^+^ cells to be distinctly angiogenic compared with CD34^+^ cells and suggest that the former subpopulation is a primary contributor to the proangiogenic properties attributed to MSCs.

## Conclusions

Here we report the isolation and characterization of CD146^+^ and CD34^+^ cell subpopulations from equine adipose tissue. The results showed that although both subpopulations display a MSC-like phenotype, CD146^+^ cells are distinctly angiogenic in vivo, indicating CD146^+^ cells maintain a pericyte phenotype during culture. Based on these results, the marker panels currently used to define MSC populations with regenerative potential may have to be reassessed, because high levels of CD146, but not the classical MSC markers, were associated with angiogenic capacity in this study. Our results also underscore the potential advantages of using defined cell populations over standard heterogeneous MSC preparations for therapy. Angiogenesis is indeed a major component of tissue repair and the use of CD146^+^ cell subpopulations may be particularly beneficial in the future to improve the efficacy of regenerative treatments both in horses and humans.

## Abbreviations

CAM: Chorioallantoic membrane
CFU-F: Colony-forming unit fibroblasts
DAPI: 4',6-Diamidino-2-phenylindole
FACS: Fluorescence-activated cell sorting
FMO: Fluorescence minus one
FSC: Forward scatter
MFI: Mean fluorescence intensity
MSC: Mesenchymal stem/stromal cells
PFA: Paraformaldehyde
SSC: Side scatter
SVF: Stromal vascular fraction
